# Hemoperitoneum after Ovulation in Systemic Lupus Erythematosus and Autoimmune Thrombocytopenia

**DOI:** 10.1155/2019/7201650

**Published:** 2019-01-16

**Authors:** Cara Buskmiller, Henri Rosenberg, Sandeep Sen

**Affiliations:** ^1^Department of Obstetrics, Gynecology, and Women's Health, St. Louis University, St. Louis, MO, USA; ^2^Department of Obstetrics and Gynecology, Icahn School of Medicine at Mount Sinai, New York, NY, USA; ^3^SSM Cancer Care at St. Mary's Health Center, St. Louis, MO, USA

## Abstract

**Background:**

Three prior cases of hemoperitoneum associated with immune thrombocytopenia (ITP) have been reported in adolescents. This reports a case of hemoperitoneum after ovulation in an adult.

**Case:**

A 34-year-old nulligravida presented with abdominal pain after a heavy period and rebound tenderness. Urine beta-hCG was negative, hemoglobin was 5.4, and platelet count was zero. CT revealed hemoperitoneum and contrast blush surrounding the left ovary. She was treated for newly diagnosed systemic lupus erythematosus and steroid-refractory ITP. Her platelet count and symptoms improved.

**Conclusion:**

Hemoperitoneum after ovulation in ITP is rare; this represents the first adult case in the literature and reviews differential diagnosis of thrombocytopenia. The obstetrician/gynecologist is part of a multidisciplinary team caring for patients with bleeding of gynecological origin and should withhold surgical intervention for hemoperitoneum when medical therapy is warranted.

## 1. Introduction

Rupture of germinal epithelium at ovulation is an ordinary and frequent occurrence in women of reproductive age, but it is not always an easy event. Three prior cases of hemoperitoneum after ovarian cyst rupture have been reported in the past in adolescents, all of which were associated with severe immune thrombocytopenia (ITP) [1–3]. This report presents a case of hemoperitoneum after ovulation in an adult, discusses the differential diagnosis, and reviews ITP, to remind the gynecologist to take care prior to offering surgical intervention for hemoperitoneum.

## 2. Case

**Figure 1:**
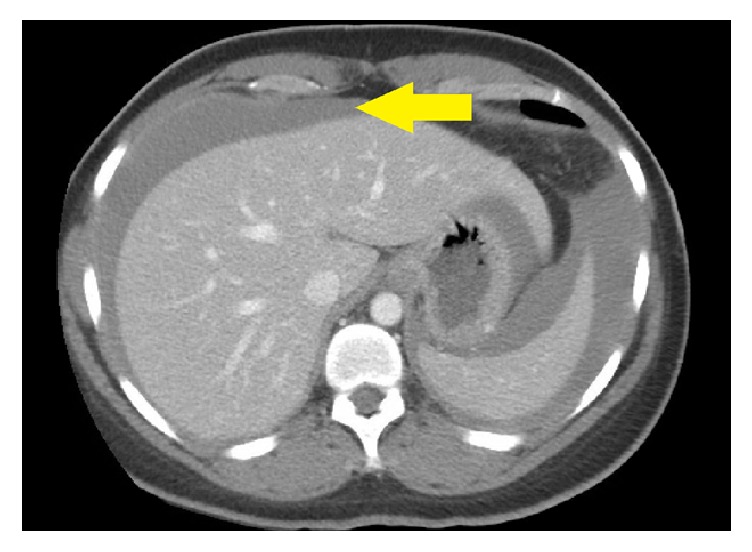
CT of the abdomen with blood surrounding the liver and spleen (arrow).

**Figure 2:**
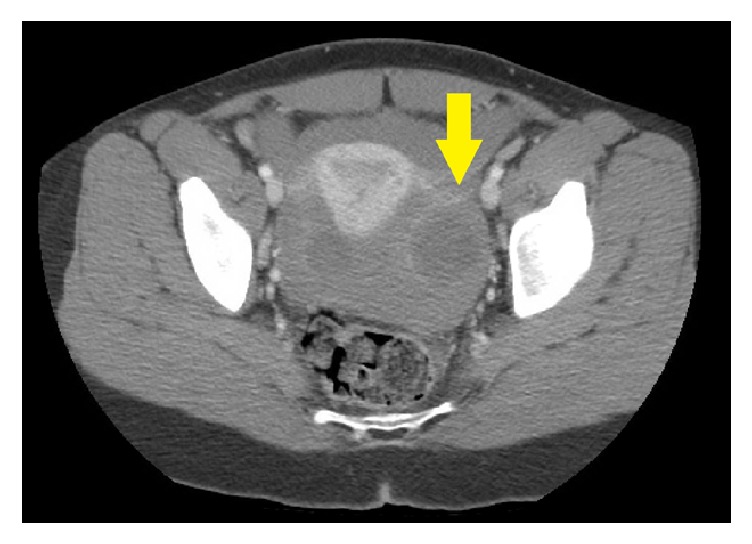
CT of the pelvis with contrast concentrated in fluid around left ovary (arrow).

A 34-year-old nulligravida with a remote history of follicular cyst treated by ovarian cystectomy presented with acute abdominal pain associated with emesis. She was hemodynamically stable, but her abdominal exam was remarkable for rebound tenderness. Complete blood count revealed hemoglobin of 5.4 mg/dL (hematocrit of 18.7%) and an undetectable platelet count. Computed tomography of the abdomen and pelvis revealed moderate-volume hemoperitoneum (Figure 1) and contrast blush surrounding the left ovary (Figure 2), which was consistent with low volume active blood loss from the left ovary.

Five weeks prior to presentation, the patient experienced prolonged gingival bleeding after a dental appointment. Two weeks following this, she began to experience spontaneous bruising, epistaxis with minimal trauma or sneezing, and cravings for ice chips. This was followed by uncharacteristically long and heavy menses, during which the patient soaked 1 pad every 1-2 hours. She began to feel fatigue and shortness of breath with minimal activity. The day prior to admission, she began to feel abdominal bloating and the following day she described waxing and waning, moderate to severe abdominal pain.

**Figure 3:**
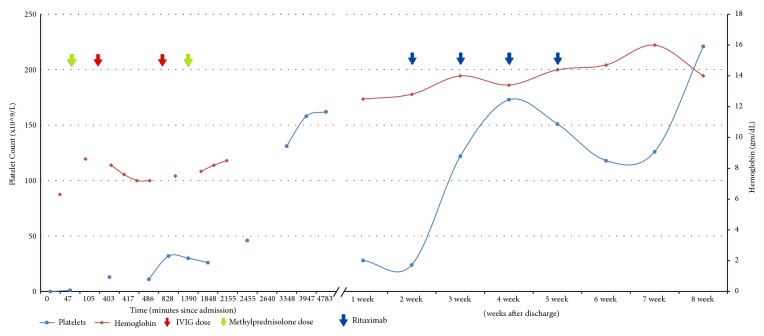
Laboratory values during the patient's course. IVIG, intravenous immunoglobulin.

She was admitted to the intensive care unit out of concern for possible spontaneous intracranial hemorrhage (ICH). She was transfused two units of platelets and two units of packed red cells; her platelet count rose only to 13 × 10^9^/L and several hours later fell to 11 × 10^9^/L (Figure 3). After review of her peripheral blood smear, hematology began empiric treatment for immune thrombocytopenia with IV immunoglobulin and IV methylprednisolone. The patient's platelet counts began to spontaneously improve without additional transfusion, consistent with a consumptive thrombocytopenia; at discharge, platelets were 162 × 10^9^/L. The patient's hemoglobin recovered appropriately after platelet count recovered, presumably because the spontaneous bleeding from the left ovary ceased.

A battery of hematologic, infectious, and rheumatologic testing revealed an antinuclear antibody (ANA) titer of 1:640, a negative double-stranded DNA (dsDNA), positive anti-Smith antibodies, positive anti-SSA antibodies, and positive anti-RNP antibodies. The patient met criteria for systemic lupus erythematosus (SLE), and her thrombocytopenia was attributed to this. Interestingly, the patient's direct Coomb's test was positive, which is unusual for ITP. The patient had normal bilirubin and liver function tests, but it was thought that she had an early synchronous autoimmune hemolytic anemia (AIHA), which can be associated with thrombocytopenia and can develop in the early years of diagnosis [4]. The patient's response to steroids and normalization of hemoglobin levels with normalization of platelet function illustrates that her AIHA was mild and responsive to steroids alone. The patient was discharged in stable condition on 1 mg/kg oral prednisone and plaquenil.

One week after discharge, the patient was without active bleeding and ecchymoses were fading. However, she was found to have a platelet count of 28 × 10^9^/L, and was treated for steroid-refractory ITP with rituximab 375 mg/m^2^ weekly for four weeks. She was placed on oral imuran in hopes of better controlling the underlying SLE. The patient responded well to these interventions, with subsequent platelet recovery to normal levels. She was vaccinated for pneumococcus and meningococcus, and a discussion was held regarding* H.* influenzae vaccination in anticipation of possible splenectomy later in life.

## 3. Discussion

This patient has a five-week history of mucosal bleeding, indicating that she was likely thrombocytopenic below 50 × 10^9^/L at that time, shortly before ovulation likely occurred based on her last menstrual period. It is suspected that she began to slowly bleed from her ovarian epithelium at ovulation and continued to build up hemoperitoneum in the intervening weeks. Symptoms beginning so acutely may be related to diaphragmatic irritation, mass effect, or overwhelming peritoneal irritation.

Severe thrombocytopenia is a rare occurrence with a wide differential diagnosis. After excluding laboratory error, possible etiologies include drug-induced or primary ITP, gestational conditions (pre-eclampsia, HELLP), disseminated intravascular coagulation, hematologic conditions (thrombotic thrombocytopenia purpura, hemolytic uremic syndrome, and aplastic anemia), hypersplenism or sequestration crisis in sickle cell disease, inherited thrombocytopenias (vWD type 2B being the most likely to present in an adult), and infections (HIV, hepatitis C, Epstein-Barr virus,* H.* pylori, intracellular parasites, and erlichiosis).

Once pregnancy is excluded and any issues of hemodynamic instability are addressed in acutely ill patients, examination of a peripheral blood smear can differentiate between mechanical and immune destruction by the presence or absence of schistocytes. Meanwhile, transfusion of platelets can be diagnostic and therapeutic: if the patient's platelet count rises appropriately, her deficiency is likely due to a productive pathophysiology; if not, it is more likely consumptive.

The differential diagnosis for bicytopenia in the setting of rheumatological workup concerning systemic lupus erythematosus should always include hematologic manifestations of lupus. While SLE and ITP are occasionally related, most ITP patients do not develop symptoms or lab findings consistent with lupus and most lupus patients do not develop thrombocytopenia which is this severe. Studies have identified different antibodies responsible for ITP-related platelet depletion and purely SLE-related platelet depletion [5], but as treatment is the same these were not clinically necessary for this patient's care.

Hematology was a key part of this patient's care and counseled the patient that the prognosis of ITP in SLE is poorer than idiopathic ITP, and the patient is at significantly greater risk of relapse and serious bleeding as a result. 9.6% of ITP patients suffer non-ICH severe bleeding, as in this case [6]. Platelet count below 10 × 10^9^/L and previous minor bleeding (such as this patient's gingival bleeding) are associated with increased likelihood of severe bleeds [6]. Prognosis of ITP is generally good: 9% of adults experience spontaneous remission, usually within the first few months; 14-40% have remission with IVIG and steroids; and the remainder require a second-line therapy, such as anti-Rh(D) (different from RhIG used in pregnancy), TPO receptor agonists (romiplostim or eltrombopag), rituximab, or splenectomy [7]. The annual risk of death from ITP per year varies from 0.4% for those younger than 40 years of age to 13% per annum in those over 60 years, and relates to bleeding complications and infections [8, 9]. Quality of life can be seriously impacted in ITP, but newer therapies are being developed, such as monoclonal antibodies targeting specific steps in thrombopoeisis and amifostine, a thrombopoeisis stimulant whose mechanism is not yet clear [8].

The patient's positive direct Coombs likely points to a mild, early autoimmune hemolytic anemia, which was thought to respond to steroids while her ITP required more aggressive therapy. The majority of her severe anemia was thought to be more likely related to intraperitoneal blood loss.

Prompt consultation with experts in hematology/oncology and other disciplines (e.g., maternal-fetal medicine, infectious disease, and rheumatology) is vital to the care of patients presenting with extremely low platelet counts and spontaneous intraperitoneal bleeding. Needless to say, these patients are usually not surgical candidates unless evacuation of hemoperitoneum is necessary for decompression of abdominal compartment syndrome.

The obstetrician/gynecologist is part of a multidisciplinary team caring for patients with bleeding of gynecological origin. When appropriate, hematology/oncology and rheumatology can be of great assistance, such as in this case of spontaneous hemoperitoneum from a bleeding ovary in the setting of SLE and ITP. It is vital that key elements of the differential diagnosis be examined prior to surgical intervention of hemoperitoneum of gynecologic origin.
